# Supplementary material for: A New Model of Learning: Environmental Health in a Global World
Source: Int J Environ Res Public Health. 2023 Jun 16;20(12):6146. doi: 10.3390/ijerph20126146 (PMC10297959; doi:10.3390/ijerph20126146)
Supplement: Supplementary file 1 [file ijerph-20-06146-s001.zip › ijerph-2436194-supplementary.pdf]

Supplementary File: A New Model of Learning: Environmental Health in a Global World

On-line Supplement: Presentation Slides

Chinese Energy: Renewables

Group 7: Hayley Ackerman (Coal Power Plants), Kujegy Kamara (Heatwaves/Flooding), Amberyn Te (Solar), Sara Amiraly (Wind Turbines), Damian Aladin (Electric Vehicles), Lucy Avant (Bullet Trains),

Coal Power Plants

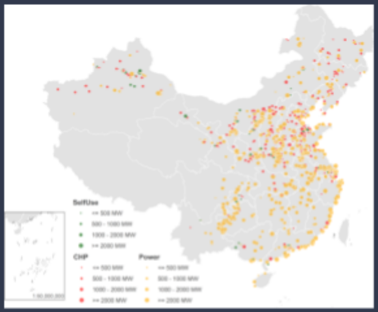

Fig 1: Location, Size and Use of Existing Power Plants in China

- China has currently one of the youngest and most robust coal-powered energy systems
  - 2021 build-up
  - 5x as much coal as India and 6x that of the USA
- President of the CCP, Xi Jinping 2020 pledge
  - Carbon neutral by 2060, peak emissions by 2030, cuts beginning in 2025
  - How has the government done so far? Challenges?
- In 2021, China was behind more than half of the new coal capacity added globally
  - China traditionally as an exporter of climate change
- How bad will it get before that 2025 benchmark?

Heat Waves and Flooding

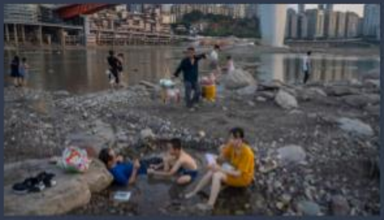

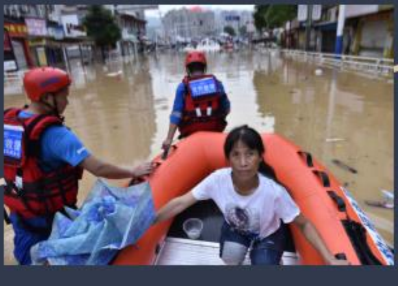

- Effects of Heat Floods on the people?
    - Unemployment has reached an all time high
    - Blackouts and factory shutdowns
    - Reduce size of harvest in China due to long periods of droughts
- Severe Drought
    - Rivers are being shrunken due to this drought, disrupting the regions supply of water which then leads to a limit of the amount of electricity being used.
    - Over 51 rivers and 24 reservoirs parched.
- Scorching Temperatures
    - For more than 70 days straight, heatwaves have ravaged throughout China with a temperature of 104 degrees, affecting about 900 million people.

Solar Panels

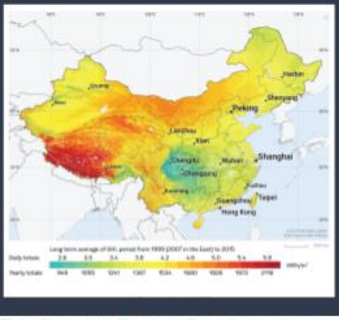

Fig 2: China's Solar Resources

Fig 3: Electricity Generation from Solar in terawatt-hours per year

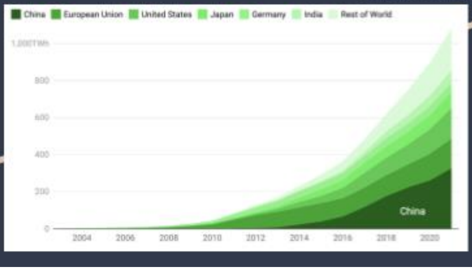

- Solar energy could provide 43.2% of China's electricity demands in 2060 at less than two-and-a-half U.S. cents per kilowatt-hour
- China has the world's largest renewable power capacity, including 323 gigawatts of solar, around a third of the entire global total
- Great potential for a global green energy transition
- Imbalances in solar supply chains
    - China's share in key manufacturing stages of solar panels -- polysilicon, ingots, wafers, cells and modules -- exceeds 80 percent, and is set to rise to more than 95 percent
- China's domination of supply chains for solar panels has come under scrutiny in some countries amid allegations of human rights abuses and trade violations
- The world will need to quadruple the pace at which solar capacity is increasing by 2030 in order to reach net-zero emissions by 2050. Global production capacity for critical components such as polysilicon, ingots, wafers, cells and modules will also need to more than double by 2030 to meet the target

Wind Turbines

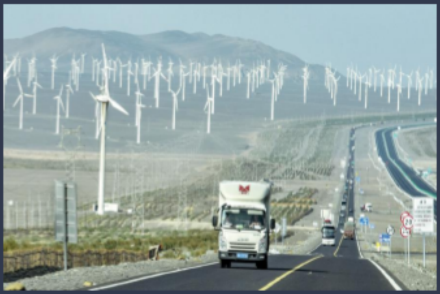

- More than 236K wind turbines in China: most wind turbines in the world
- Average cost: 2-4M $ per turbine
- Cleaner alternative to coal power plants
- Who is constructing them?
  - 3 biggest energy suppliers: Goldwind, Mingyang, and Envision Energy Ltd.
- Where are they constructed?
  - Xinjiang and Guangdong provinces
  - Displacement of people: Along with the Uyghur genocide occuring in the Xinjiang Uyghur Autonomus Region, Goldwind has been accused of forcing Uyghurs to build turbines in their factories at unlivable wages
  - Displacement of animals: Wind turbines have shown to displace animals due to habitat loss and noise pollution
- Effect on bird migration: birds avoiding wind turbine areas causes them to change migration patterns

Electric Vehicles (EVs)

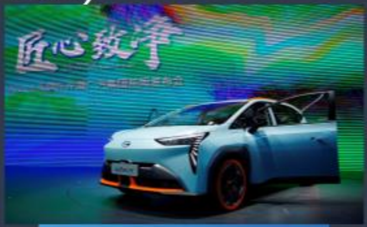

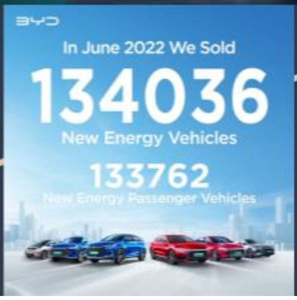

- China has the largest market for electric vehicles in the world, in terms of both buyers and manufacturers
  - Market for electric vehicles is massive in China, being sold and manufactured in a variety of prices, shapes and sizes. Prominent Chinese manufacturer BYD has a market cap about 90 billion with a 27.9% market share (Tesla at 6.6%)
  - Approximately 53% of all EVs in the world are in China, with a projected 6 million registered this year
- Purchases of EVs are encouraged through a variety of benefits and incentives
  - EV payments have been subsidized for years(slated to end 2023)
  - More the 4 million charging stations installed throughout the nation
  - Battery swapping stations for quick swaps rather than protracted charging
  - Cities like Liuzhou offer extra parking spaces and ability to drive in bus lanes
- Made possible via significant government funding and involvement, incentivizing EV production, purchases and building new infrastructure
  - Accomplished over the course of nearly a decade, other nations must catch up

Bullet Trains

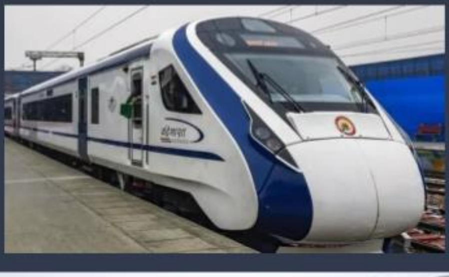

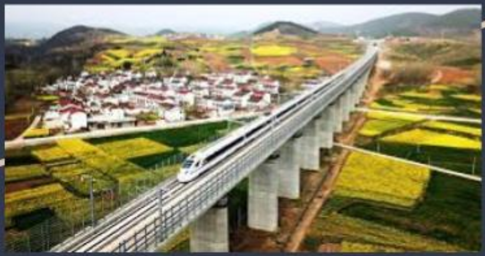

First introduced in 2007 (CRH rolling stock)

- **217 mph** using **electricity** rather than fossil fuels
- Large system
  - 2,800 pairs of trains
  - Connecting 550 cities
  - In 33/34 provinces

Impact:

- **92% less CO2** emissions than plane
- Annual **reduction of 11.2 million metric tons** of CO2 emissions

In the US…

- Efforts dating back to 1965 but no action
- Currently 3 project underway to build similar system
